# Larger First-Trimester Placental Volumetric Parameters Are Associated With Lower Pressure and More Flow-Mediated Vasodilation of the Fetoplacental Vasculature After Delivery

**DOI:** 10.3389/fphys.2020.00006

**Published:** 2020-01-24

**Authors:** Emilie Hitzerd, Igna F. Reijnders, Annemarie G. M. G. J. Mulders, Anton H. J. Koning, Irwin K. M. Reiss, A. H. Jan Danser, Régine P. M. Steegers-Theunissen, Sinno H. P. Simons, Maria P. H. Koster

**Affiliations:** ^1^Department of Pediatrics, Division of Neonatology, Erasmus MC University Medical Center, Rotterdam, Netherlands; ^2^Department of Internal Medicine, Division of Pharmacology and Vascular Medicine, Erasmus MC University Medical Center, Rotterdam, Netherlands; ^3^Department of Obstetrics and Gynecology, Erasmus MC University Medical Center, Rotterdam, Netherlands; ^4^Department of Pathology, Clinical Bioinformatics Unit, Erasmus MC University Medical Center, Rotterdam, Netherlands

**Keywords:** placental development, ultrasound, virtual reality, vascular volume, placental perfusion, vascular resistance

## Abstract

**Objective:**

To explore the correlation between *in vivo* placental volumetric parameters in the first trimester of pregnancy and *ex vivo* parameters of fetoplacental vascular function after delivery.

**Methods:**

In ten singleton physiological pregnancies, placental volume (PV) and uteroplacental vascular volume (uPVV) were measured offline in three-dimensional ultrasound volumes at 7, 9, and 11 weeks gestational age (GA) using Virtual Organ Analysis and Virtual Reality. Directly postpartum, term placentas were *ex vivo* dually perfused and pressure in the fetoplacental vasculature was measured to calculate baseline pressure (pressure after a washout period), pressure increase (pressure after a stepwise fetal flow rate increase of 1 mL/min up to 6 mL/min) and flow-mediated vasodilation (FMVD; reduction in inflow hydrostatic pressure on the fetal side at 6 mL/min flow rate). Correlations between *in vivo* and *ex vivo* parameters were assessed by Spearman’s correlation coefficients (R).

**Results:**

Throughout the first trimester, PV was negatively correlated with pressure increase (*R*_growth_ = −0.84) and, at 11 weeks GA, also positively correlated with FMVD (*R* = 0.89). At 7 weeks GA, uPVV and uPVV/PV ratio were negatively correlated with pressure increase (*R* = −0.58 and *R* = −0.81, respectively) and positively correlated with FMVD (*R* = 0.62 and *R* = 0.90, respectively).

**Discussion:**

Mainly in the early first trimester, larger placental volumetric parameters are associated with lower pressure and more FMVD in the fetoplacental vasculature after delivery. This may suggest that larger and/or more vascularized placentas in early pregnancy have better adaptive mechanisms and possibly lead to better pregnancy outcomes.

## Introduction

Placenta-related pregnancy complications, such as preeclampsia (PE) and fetal growth restriction (FGR), are highly prevalent and not only affect fetal development and pregnancy outcome, but also future maternal and offspring health (Barker et al., 2007; Bellamy et al., 2007; Steegers et al., 2010). Most of these pregnancy complications originate already in the first trimester of pregnancy (Steegers-Theunissen et al., 2013). Within this time window, development of the placental bed takes place, which is characterized by remodeling of the uterine spiral arteries, thereby creating a low-resistance circulation. Adequate remodeling is crucial to placental development, subsequently affecting embryonic and fetal health (Jauniaux et al., 2006; Burton et al., 2009). After the placental vascular network has been formed in early pregnancy, capillary growth continues until delivery, mediated by various growth factors. From mid-gestation onward, there is an exponential growth in vascular volume of fetoplacental vessels to accommodate the needs of the growing fetus (Gude et al., 2004).

Non-invasive assessment of *in vivo* placental development remains challenging, since the value of available markers of placental function and development is limited. Most commonly, placental function is assessed by the use of derivatives of the placental circulation. For example, abnormal uterine artery Doppler waveforms have been related to pregnancy complications, such as pregnancy-induced hypertension and PE (Schuchter et al., 2001; Rizzo et al., 2008). An innovative method to determine placental development resulted from the introduction of Virtual Organ Computer-aided Analysis (VOCAL), which enables the assessment of three-dimensional (3D) placental volume (PV) measurements and uteroplacental vascularisation indices (i.e., vascularisation indices, flow indices and vascularisation-flow indices). These parameters have all been associated with adverse outcomes, such as miscarriage, PE and FGR (Hata et al., 2011; Reus et al., 2013; Eastwood et al., 2017). A newly developed technique is Virtual Reality (VR) which can be combined with measurements of 3D power Doppler (PD) ultrasound volumes and visualizes the placental circulation from early pregnancy onward, in three dimensions with depth perception. As previously demonstrated by Reijnders et al. (2018) this technique is feasible and reliable for use in the first trimester of pregnancy to measure placental parameters, that reflect PV and uteroplacental vascularization of the uterine/maternal side (i.e., the placental bed).

*Ex vivo* assessment of the fetoplacental vasculature can be performed using dual-sided placental perfusion, an experimental model to study fetal vascular reactivity of a single cotyledon directly after birth. Unlike most other vascular systems, the fetoplacental vasculature is not innervated. Local vascular tone and fetal cardiac output are the main determinants of blood flow through these vessels, regulated by circulating and locally produced hormones and vasoactive compounds (Walker and McLean, 1971). Therefore, flow-mediated pressure changes in the *ex vivo* dual-sided perfused cotyledon are a measure of vascular resistance in the placenta. Jones et al. (2015) have already shown that there is a significant correlation between vascular resistance measured *in vivo* (i.e., umbilical artery Doppler velocimetry at term) and *ex vivo* placental perfusion. However, no study has yet assessed the relation between *in vivo* parameters of early placental vascular development and *ex vivo* placental vascular perfusion.

Since early non-invasive assessment of placental development is challenging and it is unknown whether available markers actually represent placental function later in pregnancy, the aim of this study was to explore whether correlations exist between *in vivo* ultrasound parameters of first-trimester placental (vascular) development and *ex vivo* parameters of fetoplacental vascular reactivity at delivery. Not only will this provide better insight in the pathophysiology of placental disorders, it could also demonstrate the need for earlier evaluation of the placental circulation.

## Materials and Methods

This explorative study was conducted within the Virtual placenta study, embedded in the Rotterdam Periconception Cohort (Predict Study), which is an ongoing prospective cohort study performed at the Department of Obstetrics and Gynecology of the Erasmus MC, University Medical Center in Rotterdam, Netherlands (Steegers-Theunissen et al., 2016). Women who participated in the Predict study before 10 weeks gestational age (GA), were also invited to participate in the Virtual Placenta study that was carried out between January 2017 and March 2018. Pregnancies conceived either spontaneously or through assisted reproduction techniques (ART) were eligible. The study protocol has been approved by the Erasmus MC Institutional Review Board (MEC 2015-494). All participating women and their partners signed written informed consent at enrolment, also on behalf of their unborn child. Women were asked for consent to use their placenta for research purposes after delivery. Women with multiple pregnancies, (gestational) diabetes, viral infections (e.g., HIV) or placental anomalies were not eligible for inclusion in this subset.

### Study Parameters

Maternal characteristics were obtained from self-reported questionnaires filled out upon enrolment. First-trimester body-mass index (BMI) and blood pressure were also measured at intake. After delivery, participating women again filled out a questionnaire on pregnancy and birth outcomes. The retrieved information was checked with data from medical records and delivery reports where available.

### Ultrasound

Participants underwent serial 3D ultrasound examinations at 7, 9, and 11 weeks GA to obtain volumes encompassing the whole embryo and placenta. Ultrasound examinations were performed by experienced sonographers only, using a Voluson E8 or E10 system (GE Medical Systems, Zipf, Austria). In the first trimester, 3D ultrasound examinations were performed using a transvaginal 6–12 MHz transducer. Vasculature of the complete placenta and embryo was imaged using PD US with standardized setting (PD gain “−8.0,” pulse repetition frequency (PRF) “0.6 kHz,” wall motion filter (WMF) “low1,” quality “high”). To minimize artifacts and measurement errors by movement, participants were asked to hold their breath for approximately 30 s during image acquisition. All ultrasound examinations were performed according to international guidelines on safe use of Doppler ultrasound in the first trimester of pregnancy (ALARA-principle) and, as such, total scanning time was kept as low as possible with a maximal duration of 30 min and a maximal thermal index of 1.3 (The British Medical Ultrasound Society, 2009; Bhide et al., 2013; World Federation For Ultrasound in Medicine and Biology, 2013; Reijnders et al., 2018).

### Offline 3D Measurements

Placental volume (PV) measurements were performed with offline specialized VOCAL software (4D View, GE Medical System) according to standardized methods, to reconstruct the trophoblast (Reus et al., 2013).

**FIGURE 1:**
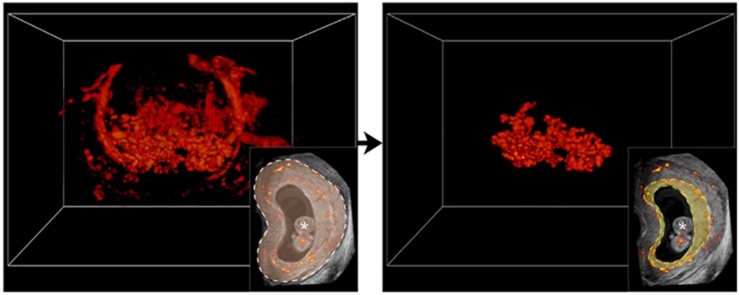
Visualization of a three-dimensional power Doppler utero-placental vascular volume in Virtual Reality. On the left; complete three-dimensional power Doppler (3D PD) vascular volume at 9 weeks of gestation. On the right; Using gray values of the utero-placental tissue, a virtual brush allows to erase vascular voxels up to the myometrial-placental tissue interface margin, leaving uteroplacental vascular volume (uPVV) to be measured by threshold-based segmentation. Lower inserts; two-dimensional power Doppler (2D PD) image with complete vasculature in color delineated by dashed white line (left), placental vascularization delineated by dashed yellow line (right). The * indicates ‘embryonic structures’.

Uteroplacental vascular volume (uPVV) was measured using a VR desktop system (Figure 1). VR enables visualization of a 3D volume as a true hologram, which allows for depth perception and thus more optimal assessment of the uteroplacental vascularization. The VR desktop is a validated system composed of a personal computer using the V-Scope volume rendering application, a two-dimensional (2D) monitor which displays the user interface, a 3D monitor to display the volume, a tracking system allowing for interaction of the observer with the 3D volume, a pair of stereoscopic glasses to enable depth perception and a six-degrees of freedom mouse for 3D volume manipulation (Baken et al., 2015). By thresholding the 8-bit (range 0–255) Doppler magnitude data, semi-automatic volume measurements of the uPVV were obtained. To enable the most optimal visualization of the uteroplacental vasculature, the lower-Doppler threshold level was set at a value of 100, which means that semi-automatic calculations only color and count by voxels with a Doppler value of 100 or higher. First, embryonic structures were removed from the segmentation. Then, the difference in echogenicity between the myometrium and placenta was used to erase the vessels up to the myometrial-placental border, thereby leaving the maternal vascularization of the uteroplacental bed for volume assessment. Currently it is not possible to distinct between the maternal blood space and embryonic vasculature within the uPVV. A more detailed description and validation of the methods for uPVV measurements has previously been published (Reijnders et al., 2018). After VOCAL and VR measurements, uPVV was divided by PV to calculate a placental vascular volume ratio (uPVV/PV ratio). Due to limited image quality or study inclusion after 7 weeks GA, measurements of PV and uPVV could not be performed for all included pregnancies and/or study visits.

### Placental Perfusion

**FIGURE 2:**
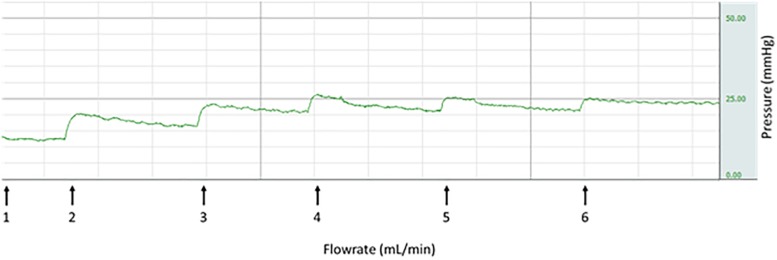
Stepwise increase in fetal flow rate leading to increase in pressure (representative).

The perfusion model used in our study has been previously described in detail by Hitzerd et al. (2019). In short, term placentas were collected immediately after delivery and fetal circulation was established by cannulating a corresponding chorionic artery and vein of an intact cotyledon. Fetal flow rate was started at 1 mL/min. When cannulation was successful, the cotyledon was cut from the placenta and placed inside the perfusion chamber. Maternal circulation (constant flow rate of 12 mL/min) was created by placing four blunt cannulas in the intervillous space. Venous outflow was collected in a reservoir underneath the cotyledon and run back to the maternal reservoir. Perfusion media consisted of Krebs-Henseleit buffer, supplemented with heparin (5000 IU, 0.5 mg/L) and aerated with 95% O_2_ – 5% CO_2_. A placental washout period of approximately 30 min was performed before starting an experiment. Changes in pressure on the fetal side of the placenta were measured by pressure transducers and recorded throughout the experiment using acquisition software (Biopac, Goleta, CA, United States). When a stable baseline pressure was reached after the washout period, the fetal flow rate was increased stepwise with 1 mL/min, until a flow rate of 6 mL/min was reached. After each step a new steady state in pressure was awaited before continuing with the next step (Figure 2). The parameters baseline pressure, total pressure increase and flow-mediated vasodilation (FMVD) were used for analysis. Total pressure increase was defined as the difference between baseline at pressure at start of the experiment and the new steady state at a flow rate of 6 mL/min. As previously described by Jones et al. (2015) FMVD is the percentage of reduction in hydrostatic pressure on the fetal side as a result of increased flow rate, measured at a flow rate of 6 mL/min.

### Statistical Analysis

Because of skewed distributions of most parameters, data are presented as medians (interquartile range). To identify correlations between *in vivo* and *ex vivo* measurements, Spearman’s rank correlation coefficients (*R*-values) were used and correlations were plotted in scatterplots. To further evaluate these correlations, linear mixed models were used to calculate individual slopes for each participant to establish placental growth trajectories throughout the first trimester (at 7, 9, and 11 weeks GA). In these models, uPVV, PV and the uPVV/PV ratio were transformed using a cubic root. The individual slopes were then also correlated with *ex vivo* parameters using Spearman’s correlation coefficients. All analyses were performed using SPSS software (version 21.0; SPSS Inc., Chicago, IL, United States) and RStudio Statistics (version 3.5.0, 2018). Correlations >0.5 were considered relevant and *P*-values <0.05 were considered statistically significant.

## Results

### Baseline Characteristics

In this explorative study, twelve women were included, of whom ten placentas were successfully perfused. Baseline characteristics of these ten women are provided in Table 1. Women had a median age of 31.9 years (29.7–37.1), 40% were nulliparous and 90% conceived spontaneously. None of the women smoked or used alcohol during pregnancy. In 60% of the women, the mode of delivery was an elective caesarean section (two nulliparous – and four multiparous women), all because a of breech position and/or previous caesarean section. Two women delivered spontaneously and another two women underwent an emergency caesarean because of failure to progress. Median birth weight was 3365 grams (2835–3425) and 70% of the offspring was male. None of the pregnancies were complicated by PE or any other pregnancy complication. All infants were born at term (i.e., >37 weeks GA), and one infant was small-for-gestational-age (birth weight below the 10th percentile) (Hoftiezer et al., 2019). Median placental weight was 395 grams (322–451), and of two placentas, weight was below the 10th percentile.

**TABLE 1:** Baseline characteristics (*n* = 10).

**Characteristics**	
**Maternal**	
Age at intake (years)	31.9 (29.7–37.1)
Nulliparous	4 (40%)
*Mode of conception*	
− Spontaneous	9 (90%)
− IVF/ICSI	1 (10%)
*Geographic origin*	
− Dutch	7 (70%)
− Western	1 (10%)
− Non-western	2 (20%)
*Educational level*	
− Low	0 (0%)
− Intermediate	3 (30%)
− High	7 (70%)
BMI, first-trimester (measured)	22.8 (21.7–32.5)
Periconceptional folic acid supplement use	10 (100%)
*Median first trimester RR (intake)*	
− Systolic	108.0 (99.0–110.0)
− Diastolic	65.0 (60.0–70.0)
Periconceptional smoking	1 (10%)
Periconceptional alcohol use	0 (0%)
**At delivery**	
Gestational age at delivery	38^+5^ (37^+4^–39^+1^)
*Mode of delivery*	
− Vaginal	2 (20%)
− Elective caesarean	6 (60%)
− Emergency caesarean	2 (20%)
Placental weight	395 (322–451)
Placental weight <p10 at birth	2 (20%)
Histology: distal villous hypoplasia	1 (10%)
**Neonatal**	
Birth weight	3365 (2835–3425)
Small-for-gestational-age	1 (10%)
*Sex*	
− Male	7 (70%)
− Female	3 (30%)

*Data are expressed as median (interquartile range) or number (percentage).*

### Placental Vascular Measurements

For the ten included pregnancies, six women had multiple measurements available for PV and eight women had multiple measurements available for uPVV. Three women had measurements available at all weeks for PV, four women had available measurements at all weeks for uPVV. A total of five measurements of PV and uPVV were available at 7 weeks GA, eight measurements were available of PV and nine of uPVV at 9 weeks GA, and five measurements were available of PV and seven of uPVV at 11 weeks GA. Median values for *in vivo* placental measurements per week GA are displayed in Table 2 and increased from a median of 3.26 cm^3^ (0.96–6.40) at 7 weeks GA to 13.36 cm^3^ (6.27–30.08) at 11 weeks GA for uPVV, a median of 20.15 cm^3^ (12.95–23.90) at 7 weeks GA to 92.76 cm^3^ (69.18–125.36) at 11 weeks GA for PV, and a median of 0.16 (0.08–0.23) at 7 weeks GA to 0.17 (0.10–0.35) at 11 weeks GA for the uPVV/PV ratio.

**TABLE 2:** Median and ranges of measurable *in vivo* placental volumetric parameters per week GA.

	**7 weeks GA (*n* = 5)**		**9 weeks GA (*n* = 9)**		**11 weeks GA (*n* = 7)**	
	***Median***	***IQR***	***Median***	***IQR***	***Median***	***IQR***
PV (cm^3^)	20.15	12.95–23.90	44.69	25.57–57.25	92.76	69.18–125.36
uPVV (cm^3^)	3.26	0.96–6.40	8.80	3.97–10.61	13.36	6.27–30.08
uPVV/PV ratio	0.16	0.08–0.23	0.16	0.14–0.33	0.17	0.10–0.35

*uPVV, uteroplacental vascular volume (in cm^3^); PV, placental volume (in cm^3^); uPVV/PV ratio, ratio between uPVV and PV; IQR, interquartile range.*

### Placental Perfusion

**FIGURE 3:**
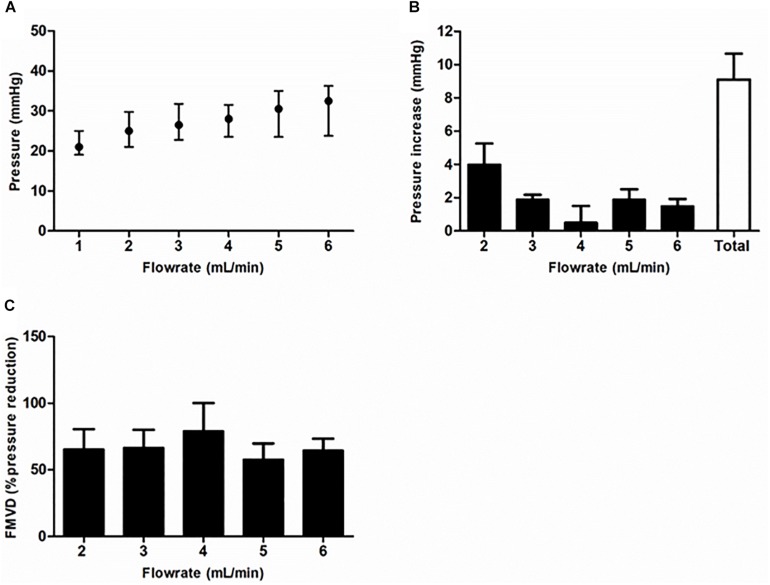
*Ex vivo* placental perfusion parameters. **(A)** Shows the steady state fetoplacental pressure that was measured after each increase of flow rate. Pressure increase was highest after the first increase of flow rate from 1 to 2 mL/min **(B)**. **(C)** Shows the flow-mediated vasodilation (FMVD) per flow rate. Data (*n* = 10) are shown as median (interquartile range).

Median GA at delivery was 38^+5^ (37^+4^–39^+1^) weeks. Median values for *ex vivo* placental measurements are displayed in Table 3. Median baseline pressure at the starting flow rate of 1 mL/min was 21 mmHg (19–25) mmHg, which increased to 32.5 mmHg (23.8–36.0) at 6 mL/min, leading to a total pressure increase of 10.5 mmHg (3.0–13.0) (Figures 3A,B). Median FMVD at 6 mL/min was 50% (50–100) (Figure 3C).

**TABLE 3:** Median and ranges of *ex vivo* placental measurements at term.

***n* = 10**	***Median***	***IQR***
Pressure at baseline	21.0	19.0–25.0
Total pressure increase (mmHg)	10.5	3.0–13.0
End pressure (mmHg)	32.5	23.8–36.0
FMVD at 6 ml/min	50.0	50.0–100.0

*FMVD, flow-mediated vasodilation (% pressure reduction from peak to new steady state); IQR, interquartile range.*

### Correlations

**FIGURE 4:**
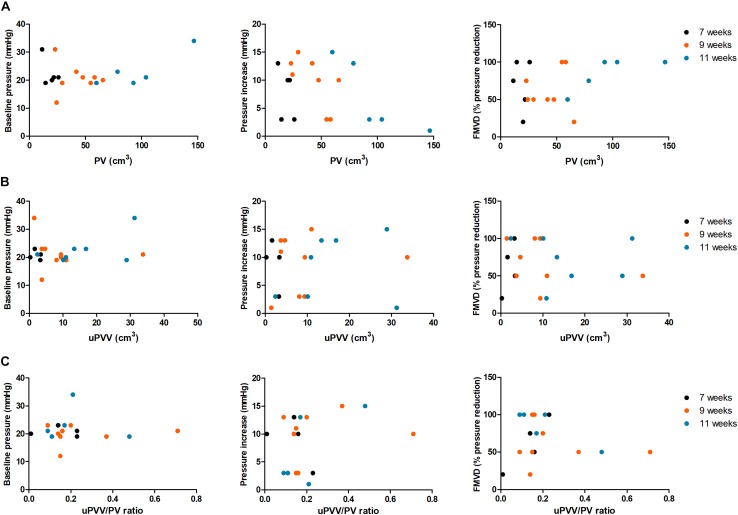
Scatterplots depicting correlations between *in vivo* and *ex vivo* placental parameters. This figure shows the correlations of the *ex vivo* parameters measured postpartum and PV **(A)**, uPVV **(B)** and the uPVV/PV ratio **(C)** at 7 weeks GA (black circles), 9 weeks GA (orange circles) and 11 weeks GA (blue circles). FMVD, flow-mediated vasodilation (% reduction in pressure from peak to new steady state); PI, pressure increase; PV, placental volume (in cm^3^); uPVV, uteroplacental vascular volume (in cm^3^); uPVV/PV ratio, ratio between uPVV and PV.

Correlations between *in vivo* and *ex vivo* measurements are depicted in Table 4 and Figure 4. PV was negatively correlated with pressure increase at 7 weeks GA (*R* = −0.53), 9 weeks GA (*R* = 0.74) and 11 weeks GA (*R* = −0.98).

**TABLE 4:** Correlations between *in vivo* and *ex vivo* placental parameters per week GA.

**Placental parameter**	**R_7__weeks_**	**R_9__weeks_**	**R_11__weeks_**	**R_growth_**
*PV*				
– pressure at baseline	–0.05	–0.05	**0.62**	0.25
– total pressure increase	**−0.53**	−0.74*	−0.98*	−0.84*
– FMVD at 6ml/min	0.05	–0.04	0.89*	**0.50**
*uPVV*				
– pressure at baseline	0.15	–0.40	0.42	0.03
– total pressure increase	**−0.58**	0.14	0.22	–0.25
– FMVD at 6ml/min	**0.62**	–0.25	–0.21	0.15
*uPVV/PV ratio*				
– pressure at baseline	–0.24	–0.01	0.05	–0.15
– total pressure increase	**−0.81**	0.12	0.36	**0.51**
– FMVD at 6ml/min	0.90*	0.21	**−0.67**	−0.90*

*R* = Spearman’s correlation coefficient. Relevant correlations in bold. *Significant at level <0.05. FMVD, flow-mediated vasodilation (% pressure reduction from peak to new steady state); uPVV, uteroplacental vascular volume (in cm^3^); PV, placental volume (in cm^3^); uPVV/PV ratio, ratio between uPVV and PV; R, correlation coefficient.

At 11 weeks GA, PV was positively correlated with baseline pressure (*R* = 0.62) and FMVD (*R* = 0.89). uPVV was negatively correlated with pressure increase (*R* = −0.58) and positively correlated with FMVD (*R* = 0.62) at 7 weeks GA. No correlations between uPVV and *ex vivo* parameters were observed at 9 and 11 weeks GA. The uPVV/PV ratio was negatively correlated with pressure increase (*R* = −0.81) and positively correlated with FMVD (*R* = 0.90) at 7 weeks GA. At 11 weeks GA, a negative correlation was observed between the uPVV/PV ratio and FMVD (*R* = −0.67), although this correlation was not statistically significant.

When studying the correlations between *in vivo* placental growth throughout the first trimester (i.e., slopes of placental parameters) and cross-sectional *ex vivo* placental parameters, a significantly negative correlation was observed between first-trimester PV growth and FMVD (*R*_growth_ = −0.84) and between uPVV/PV ratio and FMVD (*R*_growth_ = −0.90). Also, relevant positive correlations, although not statistically significant, were observed between PV growth and FMVD (*R*_growth_ = 0.50) and between uPVV/PV ratio growth and pressure increase (*R*_growth_ = 0.51).

## Discussion

The results of this study suggest that, mainly in the early first trimester, larger placental volumetric parameters, measured by 3D ultrasound and VR technique, are associated with lower pressure and more FMVD in the fetoplacental vasculature after delivery. Correlations between *in vivo* placental growth throughout the first trimester and *ex vivo* placental parameters were negative for the growth of PV and pressure increase (*R* = −0.84), but positive for FMVD (*R* = 0.50) although not statistically significant. In contrast, a negative correlation existed between the growth of first-trimester uPVV/PV ratio and FMVD (*R* = −0.90).

To our knowledge, the current study is the first to investigate associations between *in vivo* first-trimester ultrasound parameters of the maternal uteroplacental circulation and third-trimester *ex vivo* perfusion parameters of the fetoplacental circulation. Previously, it has been shown by Jones et al. (2015) that at term there is a positive correlation between *in vivo* umbilical artery Doppler velocimetry and *ex vivo* fetoplacental vascular resistance in placentas from uncomplicated pregnancies. However, one should keep in mind that umbilical artery Doppler velocimetry and *ex vivo* vascular resistance both represent the fetal side of the placenta, while our placental parameters reflect the volume and vascularization of the uterine/maternal side (i.e., the uteroplacental bed). In line with the results of Jones et al. (2015) we found a negative correlation between pressure increase at *ex vivo* perfusion and PV (throughout the first trimester) and uPVV (at 7 weeks GA only). Furthermore, this corresponds with the positive correlation that was observed between PV, uPVV and FMVD. These findings suggest a greater ability of larger placentas to adjust to higher pressure by vasodilation, due to a greater compensatory capacity in the form of vasodilation. This is contrasted by the finding that the growth trajectory of uPVV/PV ratio was positively correlated with pressure increase and negatively with FMVD, which suggests that placentas with more vascular development (i.e., more increase of uPVV compared to PV throughout the first trimester) demonstrate higher pressure and less vasodilation in response to flow. Since PV and uPVV only reflect the maternal part of the placental circulation, a possible explanation for this correlation could be that less vascularized placentas in the first trimester have been exposed to higher pressure *in utero* and therefore show more pressure increase and less vasodilation *ex vivo*.

A larger PV at 11 weeks GA was associated with higher baseline pressure in this group of uncomplicated pregnancies. In line with this, previous research by our group showed that baseline pressure during *ex vivo* perfusion in placentas of pregnancies complicated by early onset PE was significantly lower compared to healthy placentas (Hitzerd et al., 2019). These placentas were significantly smaller, exposed to higher blood pressure *in vivo*, and displayed altered vascular responsiveness. However, the direct response to flow rate increase was not studied. Interestingly, Jones et al. (2015) did not find the same positive correlation between *in vivo* umbilical artery Doppler velocimetry and *ex vivo* fetoplacental vascular resistance in placentas of pregnancies complicated by FGR as in healthy placentas. Since smaller first-trimester PV is associated with the occurrence of FGR and PE (Hafner et al., 2003), it would be interesting to study whether the correlations seen in the current study also exist in placentas of pregnancies complicated by placental insufficiency (e.g., FGR or PE). Only one patient in the current study delivered a small-for-gestational-age infant, therefore it was not possible to show a clear correlation with fetal growth, however, values of this case were not outliers. Comparing histology of the included placentas did not provide additional explanations for our results (data not shown). Histological analysis was performed according to the Amsterdam criteria (Khong et al., 2016) and included maternal stromal-vascular lesions, fetal stromal vascular lesions, infectious inflammatory lesions, immune/idiopathic inflammatory lesions, massive perivillous fibrin(oid) deposition, abnormal placental shape or umbilical insertion site, morbidly adherent placentas (accreta), meconium-associated changes and increased circulating nucleated red blood cells.

The differences in findings across the increasing gestational ages could be attributed to the unplugging of the spiral arteries around 9 weeks gestation. In early gestation, cytotrophoblast plugs occlude the spiral arteries, preventing perfusion of the intervillous space to safeguard a low-oxygen environment (Burton et al., 2003), which is needed for vasculogenesis and cytotrophoblast proliferation (Genbacev et al., 1997). Later in the first trimester, extravillous cytotrophoblast cells invade around the spiral arteries, initiating their remodeling and unplugging (Pijnenborg et al., 1980). This leads to a low-resistance circulation with an increased perfusion capacity and reduced blood flow velocity into the intervillous space (Ji et al., 2013; Roberts, 2014). We hypothesize that these vascular modifications impact PV and uPVV measurements and, especially after 9 weeks GA, could result in less pressure increase and more FMVD after delivery for larger PV and uPVV in the late first trimester. We did not demonstrate such an impact for uPVV in this study, but we did observe a negative correlation between PV and pressure increase and a positive correlation between PV and FMVD, in particular after 9 weeks GA.

This study is strengthened by the longitudinal data collection, creating a unique data set combining patient characteristics with *in vivo* and *ex vivo* measurements of the placenta. On the other hand, there is a large time gap between our measurements by first-trimester ultrasound and perfusion postpartum. Alterations in placental development during second- and third trimesters could have impacted our results, since capillary growth continues until delivery to accommodate the growing fetus, resulting in an exponential increase in volume of placental vessels in the third trimester (Gude et al., 2004). Still, it is known that failure of the maternal spiral arteries to properly remodel in early pregnancy is already associated with higher fetoplacental vascular resistance later in pregnancy (Acharya et al., 2005; Jones et al., 2015). Despite ongoing alterations, the foundation for placental vascular development is established in the first trimester, and this knowledge supports the correlations found in this study. Further, it remains uncertain whether mode of delivery could have affected vascular resistance. Most placentas in our study were obtained after elective caesarean section (70%) and have not been subjected to labor. Only two placentas were delivered vaginally and one after emergency caesarean section. There is much debate in literature whether mode of delivery affects placental perfusion experiments. On the one hand it has been demonstrated that placentas after vaginal delivery show increased oxidative stress and inflammatory cytokines on both gene- and protein levels (Cindrova-Davies et al., 2007). On the other hand, multiple studies showed no difference in placental barrier function during *ex vivo* perfusion for delivery mode (Mathiesen et al., 2010; Conings et al., 2017). Lastly, identified correlations should be cautiously interpreted due to the small sample size of the study, which also hampered correction for multiple testing. Furthermore, such small sample size could lead to bias. However, values of patients with characteristics that stood out from the rest (e.g., IVF pregnancy, periconceptional smoking, spontaneous delivery), were not outliers. Also, male/female differences could introduce bias. Unfortunately only 3 female neonates were included in this study which made verification of bias impossible, although they were not outliers. A larger sample size would have probably strengthened the identified correlations, but since this was an explorative study and *ex vivo* placental perfusion is a difficult, expanding the group size within a reasonable time frame was not feasible.

In conclusion, we showed that *in vivo* larger first-trimester PV and uPVV are associated with less pressure increase and higher FMVD of the *ex vivo* fetoplacental vasculature at term, suggesting that enhanced adaptive mechanisms after delivery relate to a more optimal development of the placenta early in pregnancy. First-trimester evaluation of PV and vascularization could therefore be of value to predict placental function in later pregnancy, thereby providing future opportunities for early prevention as well as treatment of placenta-related pregnancy complications. As a next step toward this, future research should focus on validation of these measurements in the general population and in placentas from complicated pregnancies (FGR and/or PE).

## Data Availability Statement

The raw data supporting the conclusions of this article can be made available by the authors upon request of qualified researchers.

## Ethics Statement

The studies involving human participants were reviewed and approved by the Erasmus MC Institutional Review Board. The patients/participants provided their written informed consent to participate in this study.

## Author Contributions

EH and IFR were involved in the study design, data-acquisition, performance of measurements, data-analysis, and wrote the first draft of the manuscript. AM, AK, IKR, AD, RS-T, SS, and MK were involved in the study design, data-analysis, and co-writing of the manuscript. MK  was the guarantor of this work. All authors have read and approved the final version of the manuscript.

## Conflict of Interest

The authors declare that the research was conducted in the absence of any commercial or financial relationships that could be construed as a potential conflict of interest.

## References

[B1] Acharya G. Wilsgaard T. Berntsen G. K. Maltau J. M. Kiserud T. (2005). Doppler-derived umbilical artery absolute velocities and their relationship to fetoplacental volume blood flow: a longitudinal study. *Ultrasound Obstet. Gynecol.* 25 444–453. 10.1002/uog.1880 15816007

[B2] Baken L. Van Gruting I. M. Steegers E. A. Van Der Spek P. J. Exalto N. Koning A. H. (2015). Design and validation of a 3D virtual reality desktop system for sonographic length and volume measurements in early pregnancy evaluation. *J. Clin. Ultrasound* 43 164–170. 10.1002/jcu.22207 25041997

[B3] Barker D. J. Osmond C. Forsen T. J. Kajantie E. Eriksson J. G. (2007). Maternal and social origins of hypertension. *Hypertension* 50 565–571. 10.1161/hypertensionaha.107.091512 17620523

[B4] Bellamy L. Casas J. P. Hingorani A. D. Williams D. J. (2007). Pre-eclampsia and risk of cardiovascular disease and cancer in later life: systematic review and meta-analysis. *BMJ* 335:974. 10.1136/bmj.39335.385301.be 17975258 PMC2072042

[B5] Bhide A. Acharya G. Bilardo C. Brezinka C. Cafici D. Hernandez-Andrade E. (2013). Isuog practice guidelines: use of doppler ultrasonography in obstetrics. *Ultrasound Obstet. Gynecol.* 41 233–239. 10.1002/uog.12371 23371348

[B6] Burton G. J. Hempstock J. Jauniaux E. (2003). Oxygen, early embryonic metabolism and free radical-mediated embryopathies. *Reprod. Biomed. Online* 6 84–96. 10.1016/s1472-6483(10)62060-3 12626148

[B7] Burton G. J. Woods A. W. Jauniaux E. Kingdom J. C. (2009). Rheological and physiological consequences of conversion of the maternal spiral arteries for uteroplacental blood flow during human pregnancy. *Placenta* 30 473–482. 10.1016/j.placenta.2009.02.009 19375795 PMC2697319

[B8] Cindrova-Davies T. Yung H. W. Johns J. Spasic-Boskovic O. Korolchuk S. Jauniaux E. (2007). Oxidative stress, gene expression, and protein changes induced in the human placenta during labor. *Am. J. Pathol.* 171 1168–1179. 10.2353/ajpath.2007.070528 17823277 PMC1988867

[B9] Conings S. Amant F. Annaert P. Van Calsteren K. (2017). Integration and validation of the *ex vivo* human placenta perfusion model. *J. Pharmacol. Toxicol. Methods* 88 25–31. 10.1016/j.vascn.2017.05.002 28522142

[B10] Eastwood K. A. Patterson C. Hunter A. J. Mccance D. R. Young I. S. Holmes V. A. (2017). Evaluation of the predictive value of placental vascularisation indices derived from 3-Dimensional power doppler whole placental volume scanning for prediction of pre-eclampsia: a systematic review and meta-analysis. *Placenta* 51 89–97. 10.1016/j.placenta.2017.01.005 28089506

[B11] Genbacev O. Zhou Y. Ludlow J. W. Fisher S. J. (1997). Regulation of human placental development by oxygen tension. *Science* 277 1669–1672. 10.1126/science.277.5332.1669 9287221

[B12] Gude N. M. Roberts C. T. Kalionis B. King R. G. (2004). Growth and function of the normal human placenta. *Thromb. Res.* 114 397–407. 10.1016/j.thromres.2004.06.038 15507270

[B13] Hafner E. Metzenbauer M. Hofinger D. Munkel M. Gassner R. Schuchter K. (2003). Placental growth from the first to the second trimester of pregnancy in Sga-foetuses and pre-eclamptic pregnancies compared to normal foetuses. *Placenta* 24 336–342. 10.1053/plac.2002.0918 12657506

[B14] Hata T. Tanaka H. Noguchi J. Hata K. (2011). Three-dimensional ultrasound evaluation of the placenta. *Placenta* 32 105–115. 10.1016/j.placenta.2010.11.001 21115197

[B15] Hitzerd E. Broekhuizen M. Mirabito Colafella K. M. Glisic M. De Vries R. Koch B. C. P. (2019). Placental effects and transfer of sildenafil in healthy and preeclamptic conditions. *EbioMedicine* 45 447–455. 10.1016/j.ebiom.2019.06.007 31204276 PMC6642075

[B16] Hoftiezer L. Hof M. H. P. Dijs-Elsinga J. Hogeveen M. Hukkelhoven C. Van Lingen R. A. (2019). From population reference to national standard: new and improved birthweight charts. *Am. J. Obstet. Gynecol.* 220:e1-383.e17. 10.1016/j.ajog.2018.12.023 30576661

[B17] Jauniaux E. Poston L. Burton G. J. (2006). Placental-related diseases of pregnancy: involvement of oxidative stress and implications in human evolution. *Hum. Reprod. Update* 12 747–755. 10.1093/humupd/dml016 16682385 PMC1876942

[B18] Ji L. Brkic J. Liu M. Fu G. Peng C. Wang Y. L. (2013). Placental trophoblast cell differentiation: physiological regulation and pathological relevance to preeclampsia. *Mol. Aspects Med.* 34 981–1023. 10.1016/j.mam.2012.12.008 23276825

[B19] Jones S. Bischof H. Lang I. Desoye G. Greenwood S. L. Johnstone E. D. (2015). Dysregulated flow-mediated vasodilatation in the human placenta in fetal growth restriction. *J. Physiol.* 593 3077–3092. 10.1113/JP270495 25920377 PMC4532528

[B20] Khong T. Y. Mooney E. E. Ariel I. Balmus N. C. Boyd T. K. Brundler M. A. (2016). Sampling and definitions of placental lesions: amsterdam placental workshop group consensus statement. *Arch. Pathol. Lab. Med.* 140 698–713. 10.5858/arpa.2015-0225-CC 27223167

[B21] Mathiesen L. Mose T. Morck T. J. Nielsen J. K. Nielsen L. K. Maroun L. L. (2010). Quality assessment of a placental perfusion protocol. *Reprod. Toxicol.* 30 138–146. 10.1016/j.reprotox.2010.01.006 20096346

[B22] Pijnenborg R. Dixon G. Robertson W. B. Brosens I. (1980). Trophoblastic invasion of human decidua from 8 to 18 weeks of pregnancy. *Placenta* 1 3–19. 10.1016/s0143-4004(80)80012-9 7443635

[B23] Reijnders I. F. Mulders A. Koster M. P. H. Koning A. H. J. Frudiger A. Willemsen S. P. (2018). New imaging markers for preconceptional and first-trimester utero-placental vascularization. *Placenta* 61 96–102. 10.1016/j.placenta.2017.11.013 29277277

[B24] Reus A. D. El-Harbachi H. Rousian M. Willemsen S. P. Steegers-Theunissen R. P. Steegers E. A. (2013). Early first-trimester trophoblast volume in pregnancies that result in live birth or miscarriage. *Ultrasound Obstet. Gynecol.* 42 577–584. 10.1002/uog.13197 23996572

[B25] Rizzo G. Capponi A. Cavicchioni O. Vendola M. Arduini D. (2008). First trimester uterine Doppler and three-dimensional ultrasound placental volume calculation in predicting pre-eclampsia. *Eur. J. Obstet. Gynecol. Reprod. Biol.* 138 147–151. 10.1016/j.ejogrb.2007.08.015 17916401

[B26] Roberts J. M. (2014). Pathophysiology of ischemic placental disease. *Semin. Perinatol.* 38 139–145. 10.1053/j.semperi.2014.03.005 24836825 PMC4040272

[B27] Schuchter K. Metzenbauer M. Hafner E. Philipp K. (2001). Uterine artery doppler and placental volume in the first trimester in the prediction of pregnancy complications. *Ultrasound Obstet. Gynecol.* 18 590–592. 10.1046/j.0960-7692.2001.00596.x 11844195

[B28] Steegers E. A. Von Dadelszen P. Duvekot J. J. Pijnenborg R. (2010). Pre-eclampsia. *Lancet* 376 631–644. 10.1016/S0140-6736(10)60279-6 20598363

[B29] Steegers-Theunissen R. P. Twigt J. Pestinger V. Sinclair K. D. (2013). The periconceptional period, reproduction and long-term health of offspring: the importance of one-carbon metabolism. *Hum. Reprod. Update* 19 640–655. 10.1093/humupd/dmt041 23959022

[B30] Steegers-Theunissen R. P. Verheijden-Paulissen J. J. Van Uitert E. M. Wildhagen M. F. Exalto N. Koning A. H. (2016). Cohort profile: the rotterdam periconceptional cohort (Predict Study). *Int. J. Epidemiol.* 45 374–381. 10.1093/ije/dyv147 26224071

[B31] The British Medical Ultrasound Society (2009). *Guidelines for the Safe Use of Diagnostic Ultrasound Equipment.* Available at: https://www.bmus.org/static/uploads/resources/BMUS-Safety-Guidelines-2009-revision-FINAL-Nov-2009.pdf (accessed April 25, 2017).

[B32] Walker D. W. McLean J. R. (1971). Absence of adrenergic nerves in the human placenta. *Nature* 229 344–345. 10.1038/229344a0 4925786

[B33] World Federation For Ultrasound in Medicine and Biology (2013). *Wfumb/Isuog Statement on the Safe Use of Doppler Ultrasound During 11-14 Week Scans (or earlier in pregnancy).* Available: http://www.wfumb.info/echoes-catalogue-2-2/ (accessed August 13, 2019). 10.1016/j.ultrasmedbio.2012.11.025 23398714

